# A novel *TACSTD2* mutation identified in two Chinese brothers with gelatinous drop-like corneal dystrophy

**Published:** 2009-08-14

**Authors:** Yang Jing, Chun Liu, Liya Wang

**Affiliations:** 1 Henan Key Laboratory of Keratopathy, Henan Eye Institute, Zhengzhou, PR China; 2 Department of Oncology, People’s Hospital of Dongguan, Dongguan, PR China

## Abstract

**Purpose:**

To identify the molecular defect causing gelatinous drop-like corneal dystrophy (GDLD) in two Chinese brothers and report the morphological evaluation of GDLD by laser scanning confocal microscopy and Fourier-domain optical coherence tomography (OCT).

**Methods:**

Genetic analysis included polymerase chain reaction (PCR) amplification and direct nucleotide sequencing of the coding region of the tumor-associated calcium signal transducer 2 gene (*TACSTD2*) in DNA from the two brothers and their relatives. Laser scanning confocal microscopy and Fourier-domain OCT were performed on the left cornea of the younger brother.

**Results:**

We report a novel in-frame mutation of *TACSTD2*, c.526_576del 51, in the two brothers with GDLD. The identified molecular defect cosegregated with the disease and was not found in 50 unaffected individuals. The morphological evaluation on GDLD highlighted pathological observations at the level of epithelium and anterior stroma. The epithelial cells of GDLD cornea were irregular in shape and often elongated. Large accumulations of brightly reflective amyloid material was noted within or beneath the epithelium and within the anterior stroma.

**Conclusions:**

The newly identified mutation expands the spectrum of mutations in *TACSTD2* that may cause pathological corneal amyloidosis. Observations by in vivo confocal microscopy and Fourier-domain OCT were consistent with the histopathologic descriptions of GDLD.

## Introduction

Gelatinous drop-like corneal dystrophy (GDLD; OMIM 204870) is an extremely rare autosomal recessive disease characterized by the deposition of amyloid material in the subepithelial space of the cornea. It has an estimated prevalence of 1 in 300,000 in Japan. GDLD patients have also been observed in India, the United States, Europe, Tunisia, Turkey, and Vietnam [1]. Clinical manifestations of GDLD most often appear in the first decade of life with a bilateral, axial, elevated, mulberry-like gelatinous lesion. The amyloid depositions in the central cornea, which appear in the early stage of disease, will increase in number and depth and coalesce, leading to a progressive opacification of the cornea and resulting in significant decrease in vision, photophobia, lacrimation, and foreign body sensation. Eventually, penetrating or lamellar keratoplasty, photoablation, or keratectomy is required to recover corneal clarity and visual rehabilitation. Unfortunately, symptoms generally recur within a few years after intervention, and repeated keratoplasties are often performed [2].

Genetic linkage analysis of 20 GDLD families in Japan established a 2.6 centimorgan (cM) interval on chromosome 1p as the chromosomal segment harboring the putative causal gene [3]. Later, membrane component, chromosome 1, surface marker 1 (*M1S1*), previously termed *GA733–1* or* TROP2*, was identified as the causative gene located within this region [4]. *M1S1*, in agreement with the Human Genome Organization (HUGO) nomenclature, has been renamed *TACSTD2* (tumor-associated calcium signal transducer 2) [5]. *TACSTD2* consists of a single exon spanning about 1.8 kb of genomic DNA. It codes for a protein of 323 amino acids, which is a monomeric cell surface glycoprotein expressed in the cornea, multistratified epithelia, and trophoblasts and is found in most carcinomas with high protein expression levels [4,6]. The physiologic function of *TACSTD2* has not been completely elucidated, however it is hypothesized that the protein can act as a calcium signal transducer [6].

Approximately 24 different *TACSTD2* mutations have been reported to date, predominantly in patients from Asia [7,8]. Herein, we present the clinical, histopathologic, and genetic assessment of two Chinese brothers with GDLD in which a novel *TACSTD2* mutation was identified. Our findings add to the allelic heterogeneity of this rare form of inherited corneal disease.

## Methods

This study was approved by the Institutional Review Board of the Henan Eye Institute (Zhengzhou, PR China). Informed consent in accordance with the Declaration of Helsinki was obtained from the patients and their family members who participated in this study.

### Report of cases

#### GDLD case 1

**Figure 1:**
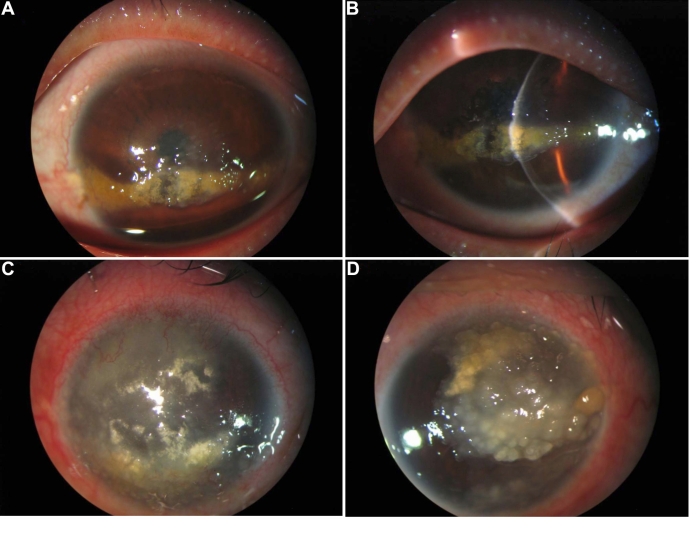
Slit-lamp photographs of affected individuals eyes. The left cornea of the younger brother show band-shaped opacities occupying the inferior cornea, consisting of yellowish deposits (**A**). Gelatinous prominences were above the band-shaped opacities (**B**). Appearance of the right cornea of the elder brother demonstrates yellowish corneal lesions with irregular surface within the entire stroma (**C**). Grayish-white nodular elevations were shown in the left cornea of the elder brother (**D**).

A 39-year-old Chinese man (the proband) was referred to our department in August 2007 with bilateral GDLD. He had complaints of defective vision since childhood. His vision had worsened in the past 10 years. He had recurrent episodes of lacrimation and photophobia in both eyes. His visual acuity was limited to counting fingers at 1 m in both eyes at the time of presentation. Under slit-lamp examination, the right cornea showed diffuse opacities with multiple grayish-white nodular elevations located in the subepithelial area whereas his left eye showed band-shaped opacities in the interpalpebral area of the cornea with several gelatinous prominences above the band-shaped opacities (Figure 1A,B). Details of the anterior chamber and lens were not visualized. In September 2007, we performed a lamellar keratoplasty on the right eye using a fresh donor cornea. At the most recent examination, the cornea showed no recurrence of GDLD with a best-corrected visual acuity (BCVA) of 20/40.

#### GDLD case 2

A 49-year-old man, the elder brother of the proband, was seen in our department in October 2007 with bilateral GDLD. He presented with the same symptoms as the proband. Visual acuity was counting fingers in the right eye and hand movements in the left eye. The patient didn’t seek medical attention until this point. Examination revealed yellowish corneal lesions with an irregular surface throughout the entire stroma and superficial vascularization in the right eye whereas his left eye showed the characteristic findings of GDLD such as multiple milky-white, opaque, semiglobate prominences (Figure 1C,D).

These two brothers had no significant systemic diseases. Ophthalmic examinations of their mother and siblings didn’t reveal any ocular abnormalities, and there were no known similar familial ocular conditions according to the report from other family members. The parents of the brothers were unaware of any consanguinity, but both parents came from the same small village. The village is isolated, and the population flow is very small.

### Mutation analysis

**Figure 2:**
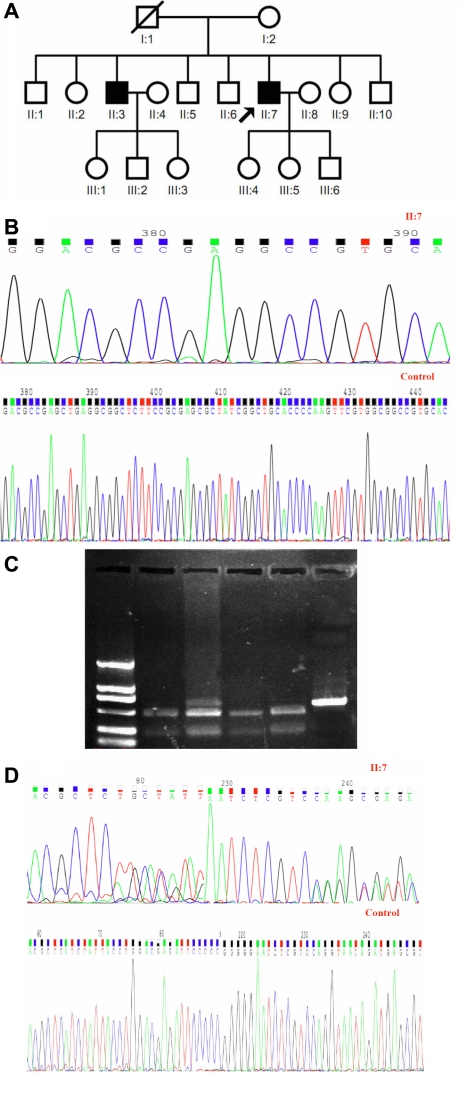
Novel *TACSTD2* mutation in the study family. **A**: Pedigree of the study family is shown. In the family tree, squares indicate male and circles indicate female. Slash denote family member who are deceased whereas heavy shading denote the individuals who are affected by GDLD. The arrow indicates the proband. **B**: The sequence from the proband, which is at the top, shows a 51 bp deletion between nucleotides 526 and 576 in the coding region of *TACSTD2*. This homozygous mutation was exclusively detected in affected family members. The normal sequence of *TACSTD2* near codon 176 is shown at the bottom. **C**: The mutation was also detected by polymerase chain reaction-restriction length fragment polymorphism (PCR-RFLP). Lane 1, Sample from unaffected control individual; lanes 2 through 4, samples from family members; lane 5, uncut amplicons. **D**: Heterozygous mutation in *TACSTD2* was detected in the study family. Sequence of the *TACSTD2* gene around codon 257 in the proband shows heterozygous 772–783del(ATCTATTACCTG)+772insT. Double-wave peaks were seen after codon 257 in sense strands or after codon 262 in antisense strands resulting from non-matching of nucleotide sequence in two alleles (top). Sequence analysis of *TACSTD2* near codon 257 in sense or codon 262 in antisense strands detected in a healthy control (bottom).

Molecular genetic analyses were performed on the two brothers and the unaffected family members (members I:2, II:1, II:5, III:2, and III:5 from this family; Figure 2A). Fifty unrelated, healthy Chinese subjects were examined as controls. Genomic DNA was obtained from peripheral blood leukocytes according to standard procedures. For amplification of the single exon of *TACSTD2*, polymerase chain reaction (PCR) was performed, and primer pairs were used as designed by Akhtar et al. [9]. Their sequences are as follows: *TACSTD2* F1: 5′-ACG TGT CCC ACC AAC AAG AT-3′, R1: 5′-CAG GTA ATA GAT GAG CGT GCG-3′; *TACSTD2* F2: 5′-GGA TGT GTC ACC CAA ATA CCA-3′, R2: 5′-CTT GAG CAG CAG ACA CTT GGA-3′; and *TACSTD2* F3: 5′-CCT ACT ACT TCG AGA GGG ACA-3′, R3: 5′-CAG GAA GCG TGA CTC ACT T-3′. The sizes of the amplified DNA fragments are 681 bp, 423 bp, and 382 bp, respectively.

Each polymerase chain reaction was performed in a 50 μl reaction mixture consisting of genomic DNA (100 ng), dNTP mixture (200 μM), 25 μl 2X GC buffer with MgCl_2_, forward and reverse primer (0.1 μM each), and 2.5 U Hotstar *Taq* polymerase (Qiagen, Hilden, Germany). GC buffer was provided by Takara Biotechnology (Dalian) Co. Ltd (Liaoning, China) and designed for amplification of templates having complex secondary structure or high GC content. Amplification reactions were performed under the following conditions: 4 min of denaturation at 95 °C followed by 30 cycles of denaturation at 94 °C for 30 s, annealing at 50−55 °C for 30 s, extension at 72 °C for 30 s, and a further extension step at 72 °C for 8 min in a Gene-Amp PCR system 9700 (Perkin-Elmer Applied Biosystems, Foster City, CA). For direct sequencing, PCR products were purified using the QIAquick PCR purification kit (Qiagen), cycle sequenced with a Big Dye Terminator cycle sequencing kit (Perkin-Elmer Applied Biosystems), and directly sequenced on both strands using an automatic DNA sequencer (ABI Prism 377 Genetic Analyzer; Perkin-Elmer Applied Biosystems) according to the manufacturer’s instructions.

Nucleotide sequences were compared with the published cDNA sequence of *TACSTD2* (GenBank accession number NM_002353.2). The novel mutation found in the *TACSTD2* coding region was confirmed by PCR-restriction fragment length polymorphism (PCR-RFLP) analysis.

### Histopathologic analysis

Corneal tissue, excised at the time of a lamellar keratoplasty in the younger brother, was available for histopathologic examination. The corneal button was sectioned and analyzed with light microscopy after staining with hematoxylin and eosin (H&E), periodic acid-Schiff (PAS), and Congo red stains.

### Confocal microscopy and Fourier-domain OCT analysis

In this study, we coupled in vivo confocal microscopy and Fourier-domain optical coherence tomography (OCT) to evaluate the morphological changes of the cornea in a patient with GDLD and compared the results with the clinical ocular surface findings and histopathologic examination.

After a detailed explanation, a laser scanning in vivo confocal microscopy with a diode laser with a wavelength of 670 nm (HRT3/Rostock Cornea Module; Heidelberg Engineering, Heidelberg, Germany) was performed on the left cornea of the younger brother. Two-dimensional confocal images of the different corneal layers were acquired. Subsequently, the left cornea of the younger brother was scanned three times with two scan patterns, the line scan and the cross-line scan, by a high-speed, high-resolution, Fourier-domain optical coherence tomography (Optovue Inc., Fremont, CA).We chose the best of the three scans for analysis.

## Results

### Mutation analysis

Two sequence variations were identified in *TACSTD2* of the brothers with GDLD. In the two brothers (case1-II:7 and case2-II:3), we found a 51 bp deletion between nucleotides 526 and 576 (526–576del 51) in coding region of *TACSTD2* (Figure 2B), which led to a loss of 17 amino acids at codons 176–192. This novel mutation was observed in the homozygous state in the affected brothers and in a heterozygous state in the proband’s mother, daughter, and nephew (I:2, III:5, and III:2). This mutation was confirmed using the restriction enzyme, BstXI (Takara Biotechnology). PCR products (681 bp) harboring the homozygous mutation were digested with BstXI. Wild amplicons were digested into 468 bp and 213 bp DNA fragments while the mutant could not be cut as the mutation erased the enzyme recognition site. Therefore, digested amplicons from patients with a homozygous mutation had only one electrophoretic band (681 bp), but those from heterozygous unaffected family members yielded 681 bp, 468 bp, and 213 bp fragments (Figure 2C).

Sequencing of the 382 bp PCR product of *TACSTD2* in the affected brothers revealed a 12 bp deletion from nucleotide position 772–783 [772–783del(ATCTATTACCTG)], resulting in a loss of four amino acids at codons 258–261. Also, in the place of the missing sequence, an insertion of T (772insT) was found. This combined mutation was confirmed by analysis of both the forward and reverse sequence data and was heterozygous in the patients and the unaffected family members (I:2, III:5, and III:2; Figure 2D).

None of the above identified mutations were detected in the control population of 50 unaffected, unrelated, healthy volunteers as determined by restriction enzyme digest.

### Histopathologic analysis

**Figure 3:**
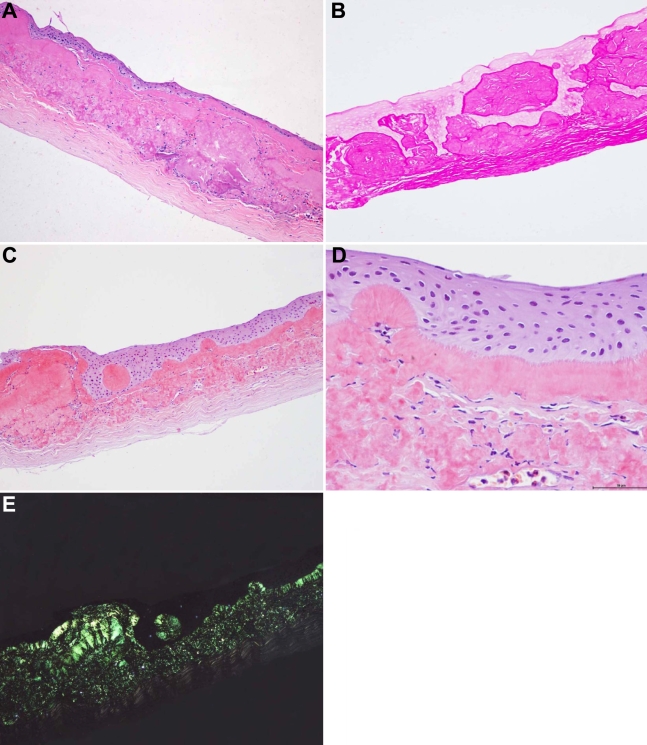
Photography of corneal histological features of the proband. **A**: A hematoxylin and eosin stained section shows epithelial atrophy, epithelial hyperplasia, and the considerable extent of the subepithelial and superficial stromal deposits of eosinophilic material. Original magnification 100X. **B**: Nodular subepithelial amorphous deposits stain positive with periodic acid-Schiff. Original magnification 100X. **C**: Congo red-positive accumulations were within or beneath the epithelium and within the anterior stroma. In some areas, Congo red-positive deposits accumulated to form gelatinous drop-like masses. Original magnification 100X. **D**: Higher magnification revealed band-like structures with a spike-like pattern just beneath the epithelium. Original magnification 400X. **E**: Congo red staining under polarized light revealed typical apple-green birefringence in epithelial and subepithelial regions, indicative of amyloid. Original magnification 100X.

Histopathologic analysis of the corneal button that was excised during a lamellar keratoplasty from the proband revealed areas of irregular epithelial hyperplasia, areas of epithelial atrophy, the destruction of Bowman’s layer, and large deposits of eosinophilic material that had positive staining of PAS and Congo red. The corneal stroma was vascularized and showed a chronic inflammatory cell infiltrate (Figure 3A-C). The deposits manifested different patterns depending on their position. Subepithelial deposits formed massive clumps that appeared to push up the epithelial layer to form mulberry-like patterns. Higher magnification of the area just beneath the epithelium revealed band-like sections with many spikes vertical to the basement line or amyloid materials. All the Congo red-positive deposits within or beneath the epithelium and within the anterior stroma manifested birefringence under polarized light (Figure 3D,E).

### Confocal microscopy and Fourier-domain OCT analysis

**Figure 4:**
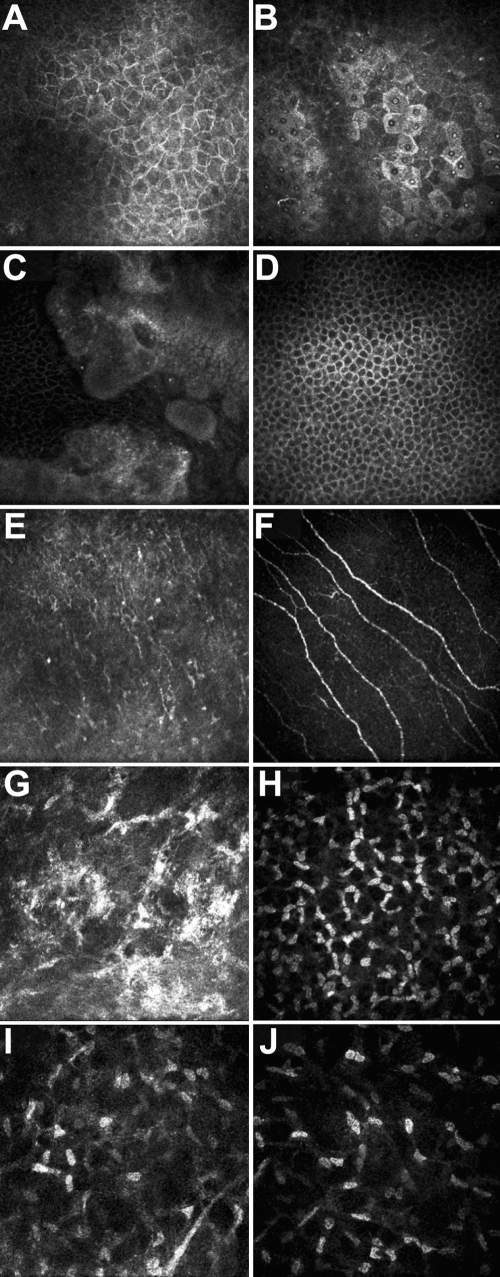
Confocal microscopy analysis of the proband’s left cornea. Superficial epithelial cells (**A, B**), wing cells (**C, D**), Bowman’s membrane (**E,F**), anterior keratocytes (**G, H**), and posterior keratocytes (**I, J**) on GDLD (**A, C, E, G, I**) and normal cornea (**B, D, F, H, J**) are displayed. **A**: The epithelial cells of GDLD cornea were irregular in shape and often elongated. **B**: Normal epithelial cells are shown. **C**: The overall epithelial architecture was destroyed. **D**: Normal wing cells are shown for comparison. **E**: At the level of the Bowman’s membrane, a very small number of sub-basal nerves were detectable. **F**: Normal Bowman’s membrane is shown. **G**: Large accumulations of brightly reflective amyloid materials were noted within the anterior stroma. **H**: Normal anterior keratocytes are shown for comparison. **I**: Posterior keratocytes of the GDLD patient. **J**: Posterior keratocytes of a normal control.

Laser scanning in vivo confocal microscopy revealed that the epithelial cells of the GDLD cornea were irregular in shape and often elongated. This was in contrast to normal epithelial cells, which appeared polygonal in form and were 30–40 µm in diameter (Figure 4A,B). Furthermore, a mild disorganization of the overall epithelial architecture was noted (Figure 4C,D). The in vivo laser scanning confocal microscopic image at the level of the Bowman’s membrane showed a very small number of sub-basal nerves with increased background intensity (Figure 4E,F). Large accumulations of brightly reflective amyloid materials were noted within or beneath the epithelium and within the anterior stroma. The nuclei of keratocytes were poorly identifiable because keratocytes were embedded in the extracellular matrix of increased reflectivity (Figure 4G,H). No evident abnormalities could be detected in posterior cornea (Figure 4I,J) .

**Figure 5:**
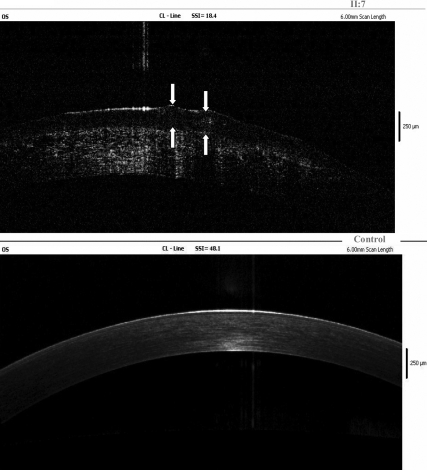
Fourier-domain OCT findings of the proband’s left cornea. Fourier-domain OCT of the proband’s left cornea showed that the main presence of amyloid deposits were localized within the epithelium and anterior stroma. The arrows are the positions of gelatinous drop-like lesions.

The images acquired from Fourier-domain OCT, showing the reflectivity of all corneal layers, indicated that the main presence of amyloid deposits was localized within the anterior stroma, extending from the epithelium layer to a depth of 150–230 µm and that the highest density of amyloid deposits seemed to be mainly in the anterior stroma, which is within the first 100 µm of the corneal depth (Figure 5).

## Discussion

The autosomal recessive form of gelatinous drop-like corneal dystrophy is a rare eye disorder caused by mutations in *TACSTD2*. Molecular genetic analysis of patients from diverse ethnic backgrounds showed both genetic and allelic heterogeneity for GDLD [10-14]. Herein, we report the molecular genetic analysis of *TACSTD2* in two Chinese brothers with GDLD in which two *TACSTD2* mutations were identified.

A 526–576del51 mutation and a 772–783del+772insT mutation were revealed by direct sequencing of the coding region of *TACSTD2* in these two brothers. The 772–783del+772insT mutation was described in a Vietnamese patient who was homozygous for this mutation [13]. In contrast, our patients and their family members (I:2, III:5, and III:2) were all heterozygous for this mutation in *TACSTD2*. Our patients are from an isolated village in the central region of China and have no ethnic relationship with the Vietnamese. However, the 526–576del51 mutation was homozygous in the GDLD-affected brothers and heterozygous in their family members (I:2, III:5, and III:2), indicating that it was cosegregated with the phenotype. Such genetic alteration was excluded in the control population. Thus, this homozygous mutation was expected to cause GDLD in the two brothers. To date, only three patients with genetically mapped GDLD were reported from China having the homozygous or compound heterozygous mutation [7,14]. The previous Chinese GDLD patient with the Q118X/Y184C mutation lived in northern China whose ethnic relationship with the Japanese was unclear. Two other GDLD patients are from two unrelated consanguineous families in the southern and southeastern regions of China. The two different mutations identified in these two patients are both homozygous frame-shift mutations, and the sites of the mutations are unique. These consisted of two insertions/deletions of a nucleotide, causing a shift in the translational reading frame and introducing a premature termination codon not far downstream of the mutation site.

The 40 kDa TACSTD2 protein contains an epidermal growth factor (EGF)-like repeat, a thyroglobulin type 1A (TY) repeat, a transmembrane domain (TM), and a phosphatidylinositol (PIP2)-binding site, harboring phosphorylatable serine and threonine residues near the COOH-terminus. The single transmembrane segment extends from position 275–298 of the amino acid sequence [4]. In our GDLD patients, the dysfunction of TACSTD2 is likely due to an in-frame deletion of 17 amino acids (L176_A192del) in TACSTD2. This in-frame deletion of TACSTD2 arises in a highly conserved region corresponding to the helix-turn-helix (HTH) configuration, and it is predicted to alter the ability of TACSTD2 to bind its target DNA sequences. The HTH is a short motif made up of an alpha-helix, a connecting turn, and a second helix, which specifically interacts with the DNA and is known as the recognition helix. The two alpha-helices extend from the domain surface and constitute a convex unit able to fit into the major groove of DNA [15-17]. Crystal structure analysis of the human TACSTD2 protein demonstrated that codons L176–A192 lie on the first alpha-helix and connecting turn of the HTH motif. Therefore, it is likely that the in-frame deletion of these codons in the two brothers interferes with the correct folding of the HTH configuration in TACSTD2. GDLD could be due to the absence of functional TACSTD2 and/or the effects of the shortened protein.

Approximately 24 different GDLD-causing mutations in *TACSTD2* have been documented to date. Most of the mutations reported have been missense and nonsense mutations. The mutations of nonsense and frame-shift are generally expected to have global detrimental effects on protein function. However, mutations causing amino acid alterations may be more informative with regard to the biochemical reason for development of a disease phenotype. With regards to GDLD, 8 of the 24 reported putative disease-causing mutations produce amino acid alterations (excluding *M1R*, which affects initiation of protein synthesis). The novel 17 amino acid deletion mutation identified in our study was within a region between the TY and TM domains, signifying the importance of these regions in relation to TACSTD2 function. The function of the region between the TY and TM domains is unknown, but the mutations in the TY domain may affect its role as a protease inhibitor and thus affect amyloid deposition [18].

Nowadays, in vivo confocal microscopy and OCT have offered useful tools in the study of normal and diseased cornea. However, no data have been published with respect to the in vivo confocal microscopy and/or OCT appearance of this disease. Our morphological evaluation on GDLD by laser scanning in vivo confocal microscopy and Fourier-domain OCT further clarifies the pathological changes of this disease. In the presented case, which is clinically diagnosed as GDLD, laser scanning in vivo confocal microscopy and Fourier-domain OCT highlighted observations at the level of the epithelium and anterior stroma, was consistent with the results of the previous histological study of GDLD [19]. However, observations obtained by these two techniques are neither completely comparable with established clinical observations by the slit-lamp biomicroscope nor with the structural descriptions of established ex vivo microscopic techniques. In summary, our data add a novel mutation identified in two Chinese brothers with GDLD to the existing spectrum of *TACSTD2* mutations.

## References

[1] Santo RM Yamaguchi T Kanai A Okisaka S Nakajima A Clinical and histopathologic features of corneal dystrophies in Japan. Ophthalmology 1995 102 557 67 7724173 10.1016/s0161-6420(95)30982-7

[2] Smolin G. Corneal dystrophies and degenerations. In: Smolin G, Tofts RA, editors. The Cornea: Scientific Foundations and Clinical Practice. 3rd ed. Boston: Little, Brown; 1994. p. 499–533.

[3] Tsujikawa M Kurahashi H Tanaka T Okada M Yamamoto S Maeda N Watanabe H Inoue Y Kiridoshi A Matsumoto K Ohashi Y Kinoshita S Shimomura Y Nakamura Y Tano Y Homozygosity mapping of a gene responsible for gelatinous drop-like corneal dystrophy to chromosome 1p. Am J Hum Genet 1998 63 1073 7 9758629 10.1086/302071 PMC1377503

[4] Tsujikawa M Kurahashi H Tanaka T Nishida K Shimomura Y Tano Y Nakamura Y Identification of the gene responsible for gelatinous corneal dystrophy. Nat Genet 1999 21 420 3 10192395 10.1038/7759

[5] Calabrese G Crescenzi C Morizio E Palka G Guerra E Alberti S Assignment of TACSTD1 (alias TROP1, M4S1) to human chromosome 2p21 and refinement of mapping of TACSTD2 (alias TROP2, M1S1) to human chromosome 1p32 by in situ hybridization. Cytogenet Cell Genet 2001 92 164 5 11306819 10.1159/000056891

[6] Ripani E Sacchetti A Corda D Alberti S Human TROP-2 is a tumor-associated calcium signal transducer. Int J Cancer 1998 76 671 6 9610724 10.1002/(sici)1097-0215(19980529)76:5<671::aid-ijc10>3.0.co;2-7

[7] Zhang B Yao YF Zhou P Two novel mutations identified in two Chinese gelatinous drop-like corneal dystrophy families. Mol Vis 2007 13 988 92 17653040 PMC2774463

[8] Alavi A Elahi E Tehrani MH Amoli FA Javadi MA Rafati N Chiani M Banihosseini SS Bayat B Kalhor R Amini SS Four mutations (three novel, one founder) in TACSTD2 among Iranian GDLD patients. Invest Ophthalmol Vis Sci 2007 48 4490 7 17898270 10.1167/iovs.07-0264

[9] Akhtar S Bron AJ Qin X Creer RC Guggenheim JA Meek KM Gelatinous drop-like corneal dystrophy in a child with developmental delay: Clinicopathological features and exclusion of the M1S1 gene. Eye 2005 19 198 204 15254496 10.1038/sj.eye.6701453

[10] Taniguchi Y Tsujikawa M Hibino S Tsujikawa K Tanaka T Kiridoushi A Tano Y A novel missense mutation in a Japanese patient with gelatinous droplike corneal dystrophy. Am J Ophthalmol 2005 139 186 8 15652848 10.1016/j.ajo.2004.06.090

[11] Ren Z Lin PY Klintworth GK Iwata F Munier FL Schorderet DF El Matri L Theendakara V Basti S Reddy M Hejtmancik JF Allelic and locus heterogeneity in autosomal recessive gelatinous drop-like corneal dystrophy. Hum Genet 2002 110 568 77 12107443 10.1007/s00439-002-0729-z

[12] Markoff A Bogdanova N Uhlig CE Groppe M Horst J Kennerknecht I A novel TACSTD2 gene mutation in a Turkish family with a gelatinous drop-like corneal dystrophy. Mol Vis 2006 12 1473 6 17167402

[13] Ha NT Chau HM Cung LX Thanh TK Fujiki K Murakami A Kanai A A novel mutation of M1S1 gene found in a Vietnamese patient with gelatinous droplike corneal dystrophy. Am J Ophthalmol 2003 135 390 3 12614764 10.1016/s0002-9394(02)01952-9

[14] Tian X Fujiki K Li Q Murakami A Xie P Kanai A Wang W Liu Z Compound heterozygous mutations of M1S1 gene in gelatinous droplike corneal dystrophy. Am J Ophthalmol 2004 137 567 9 15013888 10.1016/j.ajo.2003.08.008

[15] Pabo CO Sauer RT Transcription factors: structural families and principle of DNA recognition. Annu Rev Biochem 1992 61 1053 95 1497306 10.1146/annurev.bi.61.070192.005201

[16] Steitz TA Ohlendorf DH McKay DB Anderson WF Matthews BW Structural similarity in the DNA-binding domains of catabolite gene activator and cro repressor protein. Proc Natl Acad Sci USA 1982 79 3097 100 6212926 10.1073/pnas.79.10.3097 PMC346360

[17] Brennan RG DNA recognition by the helix-turn-helix motif. Curr Opin Struct Biol 1992 2 100 8

[18] Lenarcic B Turk V Thyroglobulin type-1 domains in equistatin inhibit both papain-like cysteine proteinases and cathespsin D. J Biol Chem 1999 274 563 6 9872988 10.1074/jbc.274.2.563

[19] Kinoshita S Nishida K Dota A Inatomi T Koizumi N Elliott A Lewis D Quantock A Fullwood N Epithelial barrier function and ultrastructure of gelatinous drop-like corneal dystrophy. Cornea 2000 19 551 5 10928776 10.1097/00003226-200007000-00029

